# The other side of the coin: systemic effects of *Serendipita indica* root colonization on development of sedentary plant–parasitic nematodes in *Arabidopsis thaliana*

**DOI:** 10.1007/s00425-024-04402-5

**Published:** 2024-04-14

**Authors:** Michael W. Opitz, Fernando Evaristo Díaz-Manzano, Virginia Ruiz-Ferrer, Roshanak Daneshkhah, Roland Ludwig, Cindy Lorenz, Carolina Escobar, Siegrid Steinkellner, Krzysztof Wieczorek

**Affiliations:** 1 Department of Crop Sciences, Institute of Plant Protection, University of Natural Resources and Life Sciences, Vienna, Tulln an der Donau, Austria; 2 Área de Fisiología Vegetal, Facultad de Ciencias Ambientales y Bioquímica, Universidad de Castilla-La Mancha, Toledo, Spain; 3 Department of Food Science and Technology, Institute of Food Technology, University of Natural Resources and Life Sciences, Vienna, Austria

**Keywords:** *Heterodera schachtii*, *Meloidogyne incognita*, Plant defense, Plant–parasitic nematodes, *Serendipita indica*, Sucrose metabolism, Sucrose synthases and invertases

## Abstract

**Main conclusion:**

Upon systemic *S. indica* colonization in split-root system cyst and root-knot nematodes benefit from endophyte-triggered carbon allocation and altered defense responses what significantly facilitates their development in *A. thaliana*.

**Abstract:**

*Serendipita indica* is an endophytic fungus that establishes mutualistic relationships with different plants including *Arabidopsis thaliana*. It enhances host’s growth and resistance to different abiotic and biotic stresses such as infestation by the cyst nematode *Heterodera schachtii* (CN). In this work, we show that *S. indica* also triggers similar direct reduction in development of the root-knot nematode *Meloidogyne javanica* (RKN) in *A. thaliana*. Further, to mimick the natural situation occurring frequently in soil where roots are unequally colonized by endophytes we used an in vitro split-root system with one half of *A. thaliana* root inoculated with *S. indica* and the other half infected with CN or RKN, respectively. Interestingly, in contrast to direct effects, systemic effects led to an increase in number of both nematodes. To elucidate this phenomenon, we focused on sugar metabolism and defense responses in systemic non-colonized roots of plants colonized by *S. indica*. We analyzed the expression of several *SUSs* and *INVs* as well as defense-related genes and measured sugar pools. The results show a significant downregulation of *PDF1.2* as well as slightly increased sucrose levels in the non-colonized half of the root in three-chamber dish. Thus, we speculate that, in contrast to direct effects, both nematode species benefit from endophyte-triggered carbon allocation and altered defense responses in the systemic part of the root, which promotes their development. With this work, we highlight the complexity of this multilayered tripartite relationship and deliver new insights into sugar metabolism and plant defense responses during *S. indica*–nematode–plant interaction.

**Supplementary Information:**

The online version contains supplementary material available at 10.1007/s00425-024-04402-5.

## Introduction

In the last years, order of Sebacinales from the phylum Basidiomycota with two phylogenetic subgroups, Sebacinaceae and Serendipitaceae, received much scientific attention (Weiß et al. 2011). These fungi are ubiquitous and ecologically diverse and form endophytic associations, in some cases mycorrhizal-like, with a wide range of plants species. They increase plant growth and development (reviewed in Franken 2012) and enhance biotic and abiotic stress tolerance (reviewed in Gill et al. 2016). *Serendipita indica* (formerly *Piriformospora indica*) is the best studied member from Serendipitaceae originally isolated in the Indian Thar Dessert from a spore of the arbuscular mycorrhizal fungi (AMF) *Funneliformis* (= *Glomus*) *mosseae* (Verma et al. 1998). *S. indica* is able to establish mutualistic relationship with *Arabidopsis thaliana* (Peskan-Berghöfer et al. 2004), where its root colonization is divided in four different stages: (1) extracellular; (2) biotrophic; (3) cell death-associated and (4) fungal reproduction (Jacobs et al. 2011). During this process, *S. indica* alters the sugar metabolism by modulating the expression of host’s *INV* and *SUS* genes and considerably changes the sugar pools in different parts of the host plant. These results demonstrated that *S. indica* seems to prefer the products of sucrose cleavage, glucose and fructose (Opitz et al. 2021). Similarly, de Rocchis et al. (2022a, b) demonstrated recently that *S. indica* influences plant carbohydrate metabolism locally (roots) and systemically (leaves) and increases sucrose phosphate synthase activity as well as resynthesis of sucrose in roots. Beside of carbohydrate metabolism, during different colonization phases, *S. indica* manipulates the expression of several genes related to plant defense and hormone signaling (Daneshkhah et al. 2018; Opitz et al. 2021), which suggests that successful root colonization by *S. indica* depends—among the others—on efficient suppression of plant immune responses.

One of the groups of important biotrophic pathogens interacting with plant roots are plant-parasitic nematodes (PPNs) being responsible for severe crop losses worldwide. Two groups, cyst nematodes (CN; *Heterodera* spp. and *Globodera* spp.) and root-knot nematodes (RKN; *Meloidogyne* spp.) are of special interest as they cause crop losses estimated at USD 80–125 billion per year (Nicol et al. 2011; Davies and Elling 2015). Those sedentary endoparasitic worms are infecting a wide range of economically important crops and the model plant *A. thaliana*. In the central cylinder, the juveniles of CN and RKN initiate the formation of sophisticated feeding sites, syncytia (Moens et al. 2018) and giant cells (Escobar et al. 2015), respectively. These feeding sites are strong sink organs supplied via massive sugar transfer from the phloem that leads to increased amounts of carbohydrates in both syncytia (Jürgensen et al. 2003; Hoth et al. 2005; Hofmann et al. 2007, 2009) and giant cells (Baldacci-Cresp et al. 2011). It was demonstrated that among the genes coding for sucrose breakdown enzymes most of *INV*s were significantly down-regulated, whereas *SUS* genes were generally up-regulated in 15-day-old syncytia (Cabello et al. 2014). Furthermore, several *SUS* and *INV* genes are regulated transcriptionally and enzymatic activity of INVs is significantly decreased in nematode feeding sites. Four genes, *VINV1*, *CINV1*, *CWINV1* and *CWINV6*, which were recently described as defective *INVs* (Le Roy et al. 2013), are responsible for this general lower activity in syncytia. Moreover, the development of both CN and RKN was significantly enhanced on multiple *sus* and *inv* mutants (Cabello et al. 2014). These results suggest that the nematodes primarily withdraw sucrose, however, excess of sugar is used for synthesis of starch as a carbohydrate buffer to compensate for changing solute uptake by the nematode during the food withdrawal phases and as long-term storage during nematode development (Hofmann et al. 2007).

Many reports demonstrated the mechanisms behind the interaction between the beneficial endophytes and biotrophic pathogens (reviewed in Schouteden et al. 2015), which seem to be equivocal and highly variable depending on the organisms and plant species (Frew et al. 2018). For instance, for RKN, Vos et al. (2012a, b) found a protective effect in different experimental set-ups in tomato showing that AMF suppresses infection and further development of *Meloidogyne incognita*. For Sebacinales, it was shown that *S. indica* antagonizes the CN *Heterodera schachtii* (Daneshkhah et al. 2013). The authors demonstrated direct effects of fungal-derived chemicals, fungal culture filtrate, and cell-wall compounds significantly inhibiting the development of *H. schachtii* in *A. thaliana*. However, it can be speculated that there is also a vast number of other factors such as antinematicidal substances, fungal volatile organic compounds, CO_2_, endophyte-triggered physiological and biochemical changes in the plant, including boost of defense responses, that affect this interaction, both directly or systemically. For RKN, it was recently demonstrated that *S. indica* promotes plant tolerance against *M. incognita* in cucumber (Atia et al. 2020). On the other hand, Frew et al. (2018) reported in wheat varieties that AMF reduces the plant biomass and suppresses compounds in roots that are related to plant resistance to migratory nematode *Pratylenchus neglectus*. Nevertheless, the endophyte was still able to provide enhanced amount of nutrients to the host. More recently, two studies also show positive effects of AMF on the potato cyst nematode *Globodera pallida* in the glasshouse (Bell et al. 2022, 2023) and the field (Bell et al. 2023). The authors showed that the majority of plant carbon was obtained by the nematodes while AMF maintained the transfer of nutrients on infected plants triggering the host tolerance against the parasite (mycorrhizal-induced tolerance, MIT) that results in increase in *G. pallida* population in soil. All those studies highlight that the AMF colonization can have positive and negative effects on different host plant traits which are important in plant–nematode interaction. These effects are highly variable and depend on environmental context as well as the taxa of both the plant and the AMF (Bennett and Bever 2007; Koricheva et al. 2009; Biere and Bennett 2013).

In conclusion, in some cases, the direct interaction between the fungal endophytes such AMF and PPNs was shown to trigger a significant reduction in nematode numbers (reviewed in Hol and Cook 2005, and Akhtar and Siddiqui 2008; Vos et al. 2012a, b, c, 2013; Daneshkhah et al. 2013). On the other hand, in some other crop plants, the AMF colonization triggered an increase in nematode populations (Frew et al. 2018; Gough et al. 2021; Bell et al. 2022, 2023). It is obvious, that the biocontrol effect depends on the AMF isolate, plant species and even its cultivar, pathogen species, type of soil and other environmental conditions (Whipps 2004). Recently, we showed that *S. indica* is able to reduce the development of PPNs in *A. thaliana* roots when both organisms occur in the same root system (Daneshkhah et al. 2013).

In this study, we aimed at investigating the interaction between *S. indica* and PPNs within the same root using split-root system where the endophyte and the nematodes are spatially separated. This should mimic the natural situation in soil where the plant root is not evenly colonized by fungal endophytes. Does the systemic *S. indica* colonization have a bioprotective effect in this particular set-up and antagonizes the nematodes as it was shown for the direct interaction? Or maybe it does enhance the development of PPNs as it was demonstrated for other fungal endophytes? To answer these questions, we analyzed the changes in plant sugar metabolism and plant defense responses as well as the systemic impact of *S. indica* colonization on development of two different endoparasitic nematodes.

## Materials and methods

### Plant materials and seed sterilization

*Arabidopsis thaliana* Col-0 was provided by Dr Alison Smith (John Innes Centre, Norwich, UK). Seeds of *A. thaliana* and *Sinapis alba* cv Albatros (P.H. Petersen, Saatzucht Lundsgaard GmbH, Grundhof, Germany) were sterilized according to Opitz et al. (2021). Briefly, surface sterilization of seeds was done for 10 min in 5% Ca(OCl)_2_ with 0.1% Tween 20 followed by 5 min incubation in 70% ethanol and three subsequent washing steps in sterile dH_2_O.

*Cucumis sativus* (L.) cv. Hoffmanns Giganta seeds (Buzzy Seeds, Pottville, PA, USA) were sterilized following the protocol by Díaz-Manzano et al. (2016). Briefly, surface sterilization of seeds was done with undiluted commercial bleach (35 g/L) for 45 min and five subsequent washing steps in sterile dH_2_O.

### Cultivation of plants

*A. thaliana* plants were grown according to Sijmons et al. (1991) in sterile Petri dishes (9 cm diameter) on Knop medium in a culture room with a 16/8 light/dark photoperiod at 23–25 °C. Plates were prepared as previously described in Opitz et al. (2021). Briefly, for analysis of direct effects, Knop medium of the “shoot area” (upper 1/3) contained 20 g L^−1^ sucrose (Knop +), whereas Knop medium of the “root area” (lower 2/3) contained no sucrose (Knop-). Onto each Petri dish, seven seeds of *A. thaliana* Col-0 were placed in line. Subsequently, plates were sealed with parafilm and put in a culture room and cultivated at the above-mentioned conditions.

**Fig. 1:**
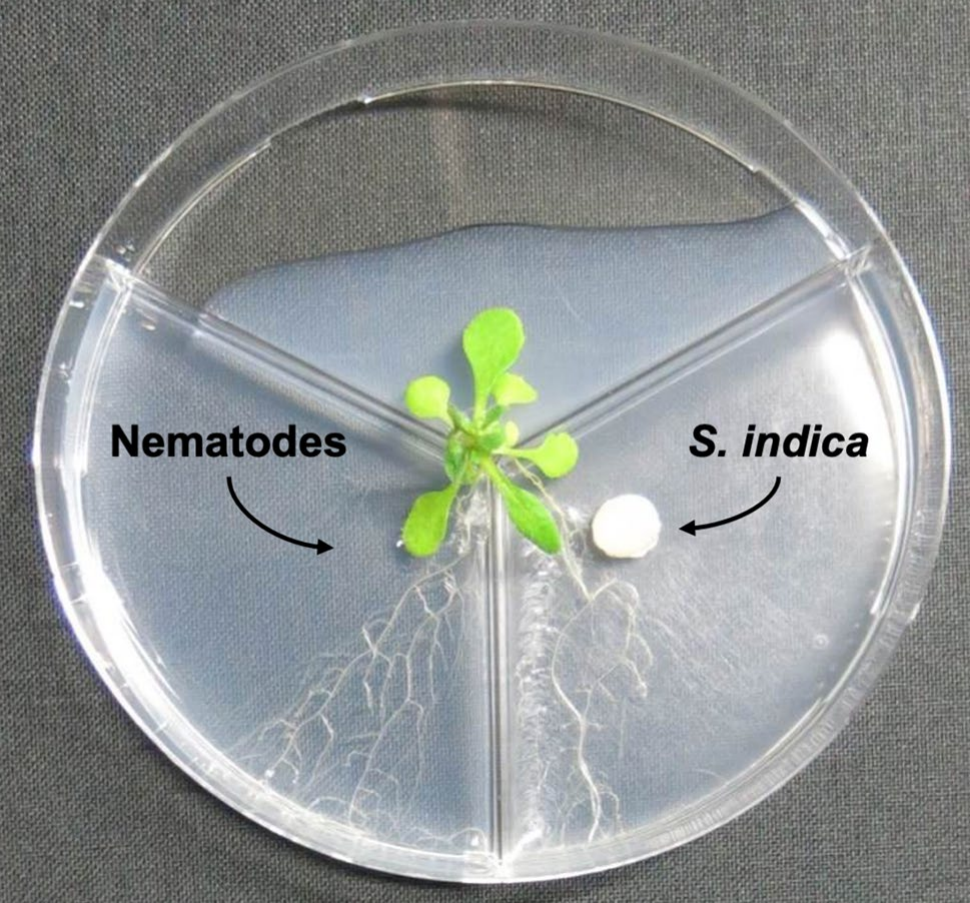
In vitro split-root system with an *A. thaliana* plant in a three-chamber Petri dish with the one half of the root system inoculated with *S. indica* and the other half infected with plant-parasitic nematodes (here the sugar beet cyst nematode *H. schachtii*)

For the systemic effect analysis, the method was adjusted to three-chamber Petri dishes (9 cm diameter). Briefly, *A. thaliana* seeds were pre-germinated in single-chamber Petri dishes containing Knop medium as described above for direct effect studies. After 4 days, main root was cut with a sterile scalpel blade inducing increased side root formation. After 5 days, single plantlets were transferred to 3-PET. Each of the two halves of the roots system was put in one Knop compartment (Fig. 1). Subsequently, plates were sealed with parafilm and put in a culture room and incubated at the above-mentioned conditions.

### *S. indica* cultivation and fungus inoculation

*Serendipita indica* (strain DSM 11827, obtained from the German Collection of Microorganisms and Cell Cultures (DSZM), Braunschweig, Germany) was provided by Prof. Ralf Oelmüller (Institut für Allgemeine Botanik und Pflanzenphysiologie, Universität Jena, Germany). The fungus was stock-cultured on Käfer’s medium following the instructions of Hill and Käfer (2001) with adaptions from Johnson et al. (2011) as previously described in Opitz et al. (2021). The fungus was pre-cultured every 4 weeks by transferring fungal plugs on new Petri dishes containing Käfer’s medium. Every 6–12 months, the fungus was co-cultivated and re-isolated from *A. thaliana* roots. Experimental plates were inoculated with *S. indica* plugs of 6 mm diameter excised from 4-week-old *S. indica* culture grown on Käfer’s medium. For analysis of direct effects, two plugs were placed top side down next to the root tips. For analysis of systemic effects, one plug was placed upside down next to root tips in one of the two compartments. Control plates were inoculated with the same number of Käfer’s medium plugs without the fungus.

### Nematode stock cultures and infection assays

All work (including maintenance of stock culture) with the sugar beet cyst nematode *Heterodera schachtii* (BCN; Sijmons et al. 1991) was performed following the instructions of Bohlmann and Wieczorek (2015). Briefly, *S. alba* was cultured in Petri dishes (14.5 cm diameter) containing Knop + medium at room temperature in the dark. BCN infection with freshly hatched J2s was performed on 14-day-old seedlings. Two months after infection, developing cysts were harvested with a tweezer and transferred into a 100 µm hatching funnel containing sterile dH_2_O. Subsequently, cysts were sterilized for 10 min in 5% Ca(OCl)_2_ containing 0.1% Tween 20 and washed four times in sterile dH_2_O. Hatching was stimulated by transferring the cysts into 3 mM ZnCl_2_ solution. Additional sterilization of hatched J2s was performed for 2 min in 0.05% HgCl_2_ solution using a 15 µm sieve. After washing 4 times in sterile, dH_2_O J2s were collected in a block dish and excess water was removed. J2 concentration was adjusted by adding 0.7% gelrite (Duchefa, RV Haarlem, Netherlands).

For analysis of systemic effects, one half of the root system was inoculated with *S. indica* as described above. Control plates were inoculated with Käfer’s medium plugs without the fungus. Prior BCN infection, length of the roots was estimated according to Jürgensen (2001). Plates were inoculated with ~ 50 freshly hatched J2s. Development of females and males was determined 14 days after inoculation (dai; BCN). Cyst size as well as length and size of corresponding syncytia was measured using inverted microscope (Axiovert200M; Zeiss, Hallerbergmoos, Germany) with an integrated camera (AxioCam MRc5; Zeiss) and AxioVision software.

The root-knot nematode *Meloidogyne javanica* Treub [1885; identified by morphology, isozymes pattern and specific primers by PCR described in Robertson et al. (2009)] was stock-cultured on roots of *Cucumis sativus* (L.) cv. Hoffmanns Giganta seeds (Buzzy Seeds) grown on modified Gamborg B5 solid medium (supplemented with 3% sucrose) following the protocol by Díaz-Manzano et al. (2016). Five seeds of *C. sativus* were sawn per Petri dish (14 cm diameter). Plates were kept at 26 °C in the dark for root system development. RKN infection was performed with ~ 1000 sterile J2s. Plates were sealed with parafilm twice, covered with aluminum foil and placed back into the growth chamber for further 2 months.

Prior to the infection assays, J2s were hatched from 25 to 50 sterile egg masses. Egg masses were obtained from stock culture plates and were placed into a sterile beaker containing a 70 µm cell strainer (Thermo Fisher Scientific, Wilmington, NC, USA) and 2.5–5 mL of sterile tap water. Hatching occurred for 4 days at 26 °C in the dark. Freshly hatched J2s were collected by pipetting into sterile centrifuge tubes with conical bottom. Excess water was removed and collected juveniles were used for infection. One day before infection, the length of the roots was estimated according to Jürgensen (2001). Plates prepared for the analysis of direct effects were infected with 15 juveniles per plant, whereas plates for analysis of systemic effects were infected with 45 juveniles per plant due to bigger root system. For better infection, a thin layer of warm but still liquid Knop media was pipetted above the inoculated root area as soon as the liquid phase of the added J2 solution was absorbed (usually 30–60 min). Plates were sealed and placed back into the growth chamber at above mentioned conditions. Plates were covered with several thin layers of cloth reducing light intensity after inoculation.

Direct effects on the number of developing RKN as well as the gall size were determined at 10 dai (RKN) using SZX16 stereo microscope (Olympus, Japan, Tokyo) with DP73 microscope camera (Olympus) and the FIJI software (Schindelin et al. 2012).

### Sugar pool analysis

For carbohydrate extraction, systemic roots and shoots of *A. thaliana* Col-0 colonized by *S. indica* as well as their uncolonized controls were harvested at 3, 7 and 14 dai (*S. indica*), ground with Mixer Mill MM 400 (Retsch GmbH, Haan, Germany) for 1–3 min at 30 Hz and stored at − 80 °C. Samples consisting of fine ground frozen plant tissue were subjected to soluble carbohydrate extraction according to Leach and Braun (2016) with adaptions from Srivastava et al. (2008). The extraction procedure, subsequent sample preparation as well as analysis by ion chromatography with pulsed amperometric detection were performed as described in Opitz et al. (2021).

### RNA isolation and cDNA synthesis

Systemic roots and shoots of *A. thaliana* Col-0 colonized by *S. indica* as well as their uncolonized controls were harvested at 3, 7 and 14 dai (*S. indica*), ground with Mixer Mill MM 400 (Retsch GmbH) for 1–3 min at 30 Hz and stored at − 80 °C. RNA extraction was done according to manufacturer’s instructions using RNeasy Plant Mini Kit (Qiagen, Hilden, Germany) including on-column DNA digestion using RNase-Free DNase Set (Qiagen). RNA concentration and purity were assessed using Nano Drop 2000c (Thermo Fisher Scientific). Samples were adjusted to 100 ng RNA µL^−1^. cDNA synthesis was done using Invitrogen SuperScript III reverse transcriptase (Thermo Fisher Scientific) according to manufacturer’s instructions. All steps were performed as described in Opitz et al. (2021). Briefly, each sample contained 22 µL RNA, 2 µL dNTPs, 2 µL of hexa oligos and 2 µL of ddH_2_O. The mixture was heated for 5 min at 65 °C followed by a 1 min incubation on ice. Subsequently, to each sample 8 µL 1st strand buffer, 2 µL 0.1 M DTT, 1 µL RiboLock RNase inhibitor (Thermo Fisher Scientific) and 1 µL reverse transcriptase were added. Samples were incubated for 5 min at 25 °C, before cDNA synthesis was performed for 60 min at 50 °C using a gradient Mastercycler (Eppendorf). cDNA synthesis was stopped by a heating step for 15 min at 75 °C. Samples were stored at -20 °C until further use.

### qPCR

qPCR was done using a peqSTAR 96Q Real Time-PCR-Cycler (Peqlab Biotechnologie GmbH, Erlangen, Germany). Reactions were performed according to manufacturer’s instructions using KAPA SYBR^®^ FAST qPCR Master Mix (2 ×) kit (Merck) containing KAPA SYBR FAST DNA Polymerase, reaction buffer, dNTPs, SYBR Green I dye and MgCl_2_ at a final concentration of 2.5 mM as described in Opitz et al. (2021). Briefly, for each gene a master mix with gene-specific primers was prepared containing 7 µL ddH_2_O, 10 µL KAPA SYBR^®^ FAST qPCR Master Mix (2×) kit, 0.5 µL of 10 mM 5′ primer and 0.5 µL of 10 mM 3′ primer. Regarding carbohydrate metabolism, six genes coding for sucrose synthases (*AtSUS1*, *AtSUS2*, *AtSUS3*, *AtSUS4*, *AtSUS5*, *AtSUS6*) and two cytosolic invertases (*AtCINV1*, *AtCINV2*) were analyzed. Impact on plant defense was assessed studying marker genes *AtEIN3*, *AtERF1*, *AtPDF1.2*, *AtOXI1*, *AtACS6*, *AtPR3* and *AtBI1*. As an endogenous control, *AtUBP22* was used (Hofmann and Grundler 2007). Sequences of primers used are shown in Supplementary Table S1. Each reaction contained 2 µL of respective cDNA. qPCR was done as follows: initial denaturation step (20 s at 95 °C) followed by 40 PCR cycles (15 s at 95 °C and 20 s at 60 °C) and a melting stage. The relative fold changes in gene expression were calculated based on the comparative CT method (Schmittgen and Livak 2008) using software peqSTAR96Q V2.1 (Peqlab Biotechnologie GmbH).

### Statistics

Statistical analysis was done using SPSS statistics software version 24.0 (Ehningen, Germany) and SigmaPlot version 14.0. Sugar pool analysis was performed in three independent replicates (*n* = 3) with pooled plant material from 36 plants each. Differences between time points were assessed for each carbohydrate separately, using One-way ANOVA (Tukey’s test, *P* < 0.05). For fructose and raffinose values in systemic roots of colonized plants, Welch ANOVA was performed (Dunnett-T3 test, *P* < 0.05). Sugar pool ratio was analyzed using Student’s *t* test at *P* < 0.05.

Infection tests with BCN were performed in three independent biological replicates (*n* = 3) with 162 plants in three-chamber Petri dish in total. Differences in infection are presented as a percentage of infection number observed in *S. indica*-colonized plants in comparison to uncolonized control plants using Student’s *t*-test (*P* < 0.05). A total of 132 root segments were collected, differences in cyst size, syncytia size and syncytia length between *S. indica*-colonized and uncolonized control plants were compared using Student’s *t*-test (*P* < 0.05).

Infection tests for direct effects with RKN were performed in three independent biological replicates (*n* = 3) in 35 plates and a total of 245 plants. Values were represented as a percentage of *S. indica* colonized plants in comparison to control plants. Infection tests for systemic effects with RKN were performed in four independent biological replicates (*n* = 4) from a total of 89 infected plates and plants. The RKN numbers were normalized to the number of root tips present at the time point of inoculation. Differences in infection between *S. indica*-colonized and uncolonized control plants were calculated using Student’s *t*-test (*P* < 0.05). Differences in gall size in roots of *S. indica*-colonized and uncolonized control plants were calculated using Student’s *t*-test (*P* < 0.05), for direct effect study (*n* = 58) and systemic effect study (*n* = 110), respectively.

qPCR was performed in three independent biological replicates (*n* = 3) with pooled plant material from 8 to 12 plants each. All measurements were performed with three technical replications. Differences in gene expression compared to the respective control were assessed using Student’s t-test (*P* < 0.05).

## Results

### Sugar levels and expression of *AtSUS *and *AtCINV* genes in systemic roots of *S. indica*-colonized plants

**Fig. 2:**
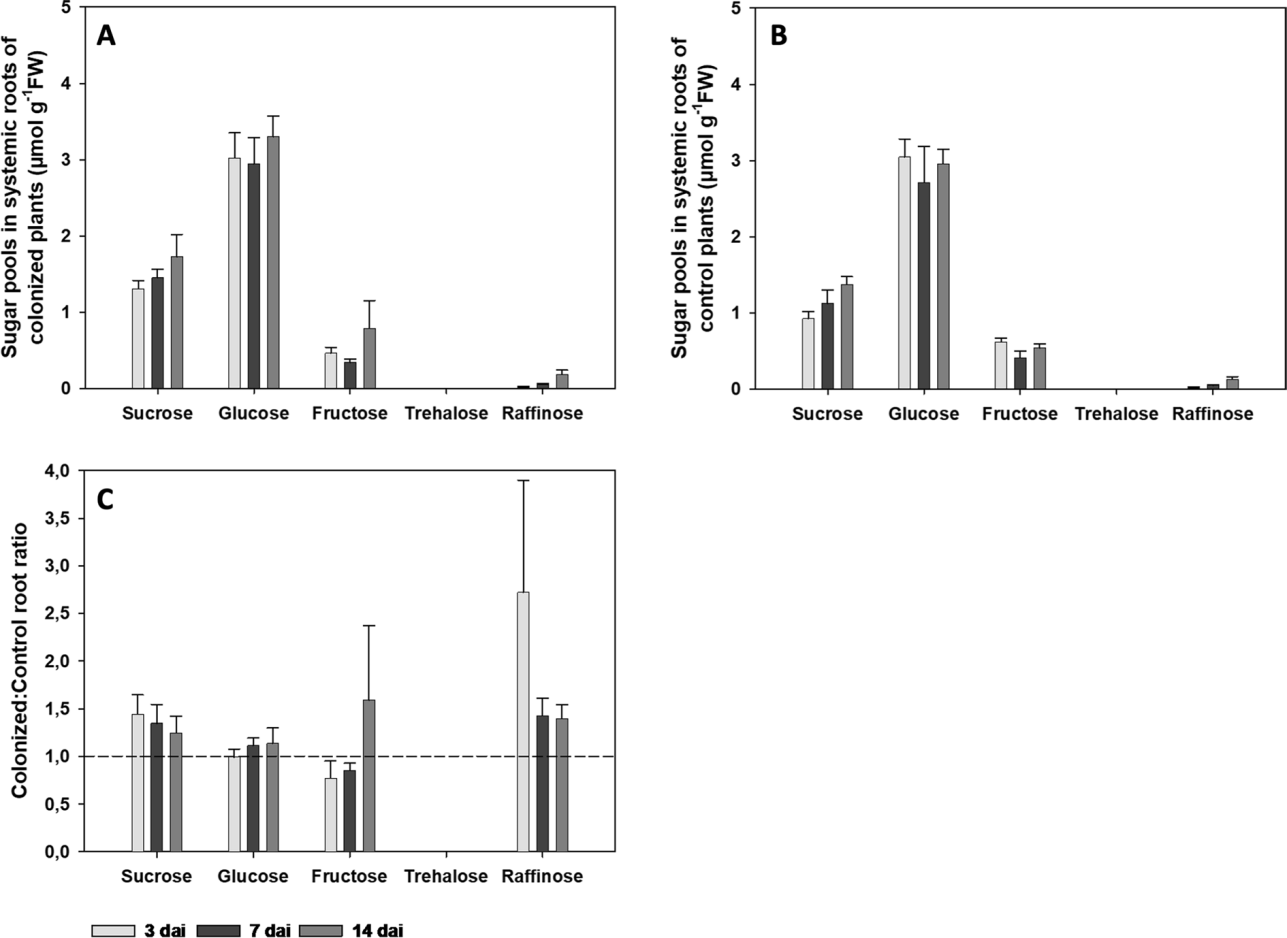
Sugar pool analysis of systemic roots of *A. thaliana* 3, 7 and 14 days after inoculation (dai) with *S. indica*. **A** Sugar pools in systemic roots of plants colonized by *S. indica*. **B** Sugar pools in systemic roots of control plants. **C** Sugar pool ratios of systemic roots of *S. indica*-colonized plants to non-colonized control roots of *A. thaliana*. Values indicate means ± SE of three independent repetitions from pooled plant material of 12–24 plants each. Differences between time points were assessed for each carbohydrate separately, using One-way ANOVA (Tukey’s test, *P* < 0.05) and are statistically non-significant. In case of fructose and raffinose values in systemic roots of colonized plants Welch ANOVA was performed (Dunnett-T3 test, *P* < 0.05). For sugar pool ratios the differences were analyzed using Student’s t test at *P* < 0.05 and are statistically non-significant

No significant differences were observed in concentration of sucrose, glucose, fructose and raffinose between the analyzed timepoints, neither in systemic roots of *S. indica*-colonized plants (Fig. 2A), nor in systemic roots of control plants (Fig. 2B). For sucrose and raffinose, the concentration increased over time with highest values in both systemic roots of *S. indica*-colonized plants as well as in systemic roots of control plants at 14 dai (*S. indica*) (Fig. 2A, B). Glucose and fructose did not show any changes in their concentration in systemically colonized and control roots at any timepoint.

The comparison between systemic roots of *S. indica*-colonized plants and systemic roots of control plants shows an increase of the sucrose and raffinose ratio towards systemic roots of colonized plants as compared to their respective controls (Fig. 2C). There was no difference in glucose ratio between *S. indica* colonized and systemic roots of control plants at any timepoint. In the case of fructose, an increased ratio is visible only at 14 dai (*S. indica*). The raffinose ratio is however insignificantly increased at 3 dai, at 7 and 14 dai.

**Fig. 3:**
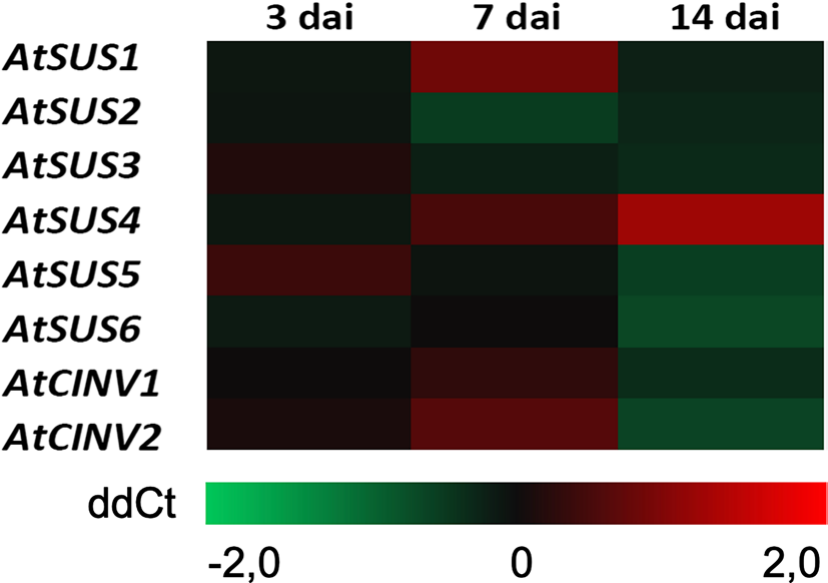
Gene expression of *AtSUS* and *AtCINV* genes in systemic roots of *A. thaliana* plants colonized by *S. indica* in comparison to non-colonized control plants at 3, 7 and 14 dai. Colors represent means of three biological independent repetitions. ddCt values are shown in Supplementary Table S2

To test whether there is a correlation between sugar pools and the expression of *AtSUS* and *AtCINV* genes in systemic roots of *S. indica*-colonized and systemic roots of control plants at 3, 7 and 14 dai, qPCR analyses were performed. These results demonstrate no significant differences in expression of these genes (Fig. 3, Supplementary Table S2).

### *S. indica* colonization affects differently the development of cyst- and root-knot nematodes

**Fig. 4:**
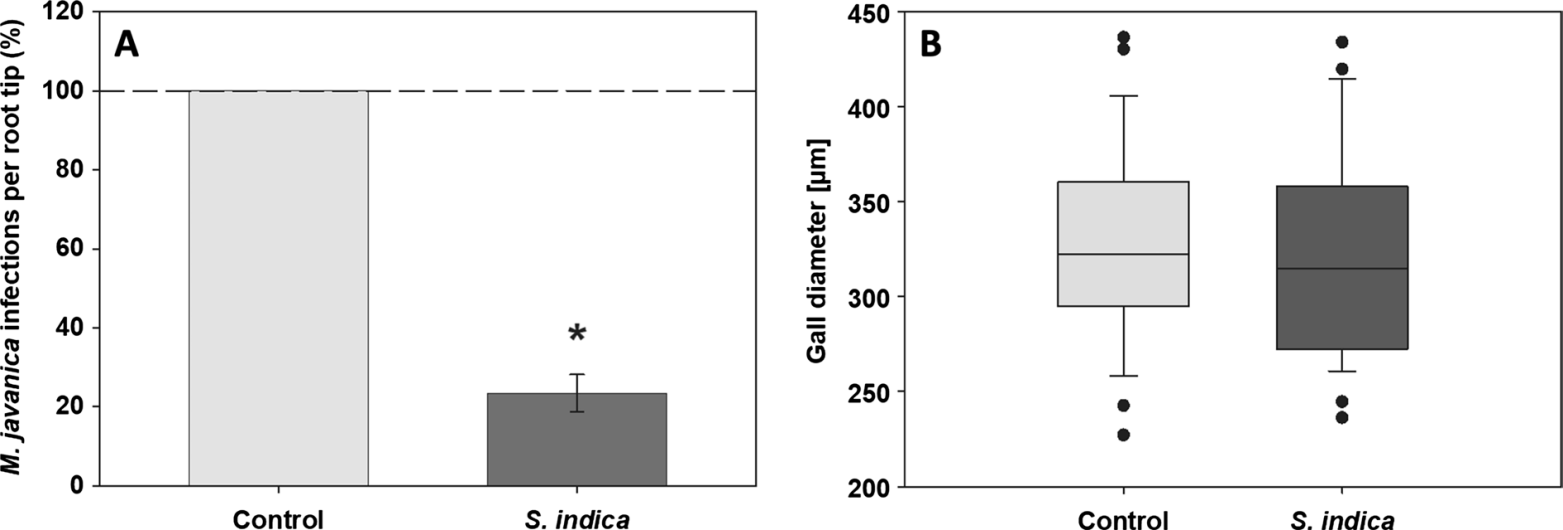
The direct impact of *S. indica* colonization on the development of root-knot nematode *M. javanica* (RKN). **A** Number of RKN galls in roots of non-colonized and *S. indica-*colonized *A. thaliana* at 10 dai (RKN) (*n* = 245). Significant reduction in the number of galls in plants colonized by *S. indica* (*P* < 0.05). Values indicate means ± SE from three independent experiments. Differences were analyzed using Student’s *t* test (*P < 0.05). **B** Diameter of RKN galls at 10 dai (*n* = 58) in plants colonized by *S. indica* and non-colonized plants. Values indicate means ± SE from three independent repetitions. Differences were analyzed using Student’s t test (*P* < 0.05) and are statistically non-significant

**Fig. 5:**
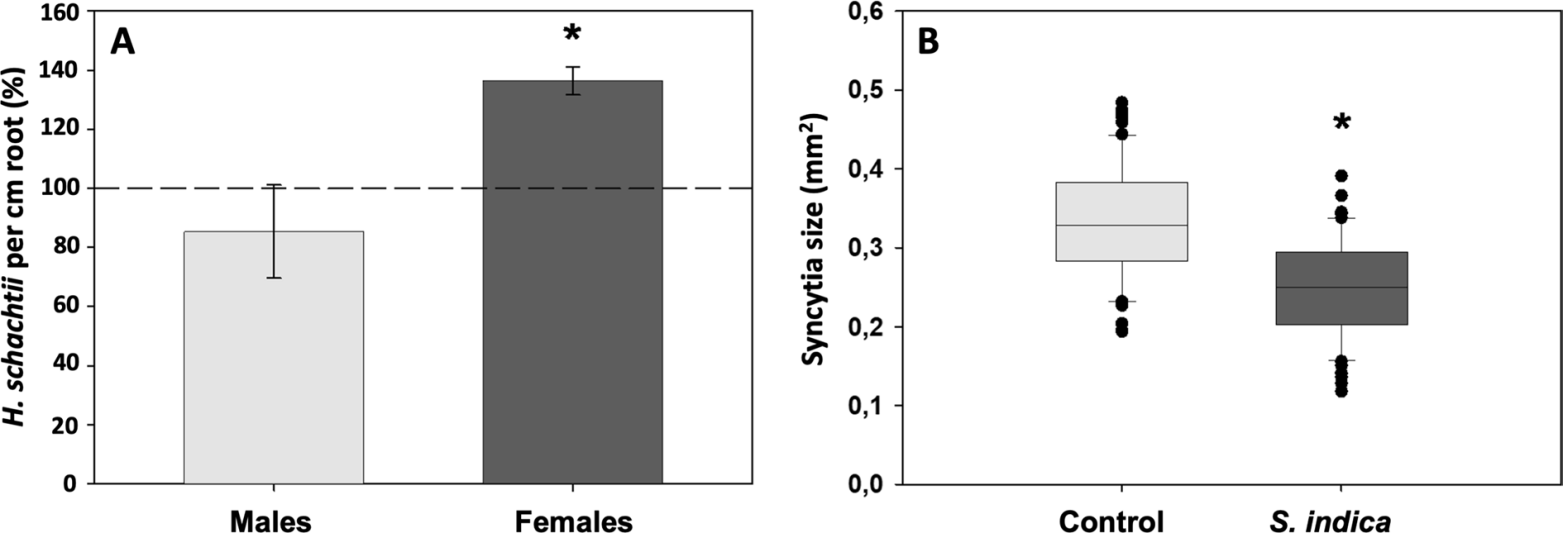
The indirect impact of *S. indica* colonization in split-root system on the development of the cyst nematode *H. schachtii* (BCN). **A** The number of BCN males and females in systemic roots of *S. indica-*colonized *A. thaliana* roots at 14 dai (BCN) (*n* = 162). Results are shown as percentages of the systemic roots of *S. indica-*colonized with *S. indica* relative to the non-colonized control plants. Values indicate means ± SE of three independent biological replicates. **B** Size of BCN*-*induced syncytia (*n* = 132) in systemic roots of *S. indica-*colonized *A. thaliana* roots 14 dai (BCN). Differences were analyzed using Student’s *t* test (*P < 0.05)

Fungal colonization at 10 dai (RKN) significantly reduces the development of RKN (Fig. 4A; − 76.6%). However, the size of galls does not significantly differ between the systemic roots of both *S. indica*-colonized and control non-colonized plants (Fig. 4B). In contrast, at 14 dai (BCN) in systemic roots of *S. indica*-colonized plants, the number of BCN females increased significantly (Fig. 5A, + 36.4%), whereas the male number was not affected. Interestingly, the size of syncytia induced in systemic roots of *S. indica*-colonized plants decreased significantly (Fig. 5B). In contrast, the length of syncytia and cyst size showed no differences in comparison to the control (data not shown).

**Fig. 6:**
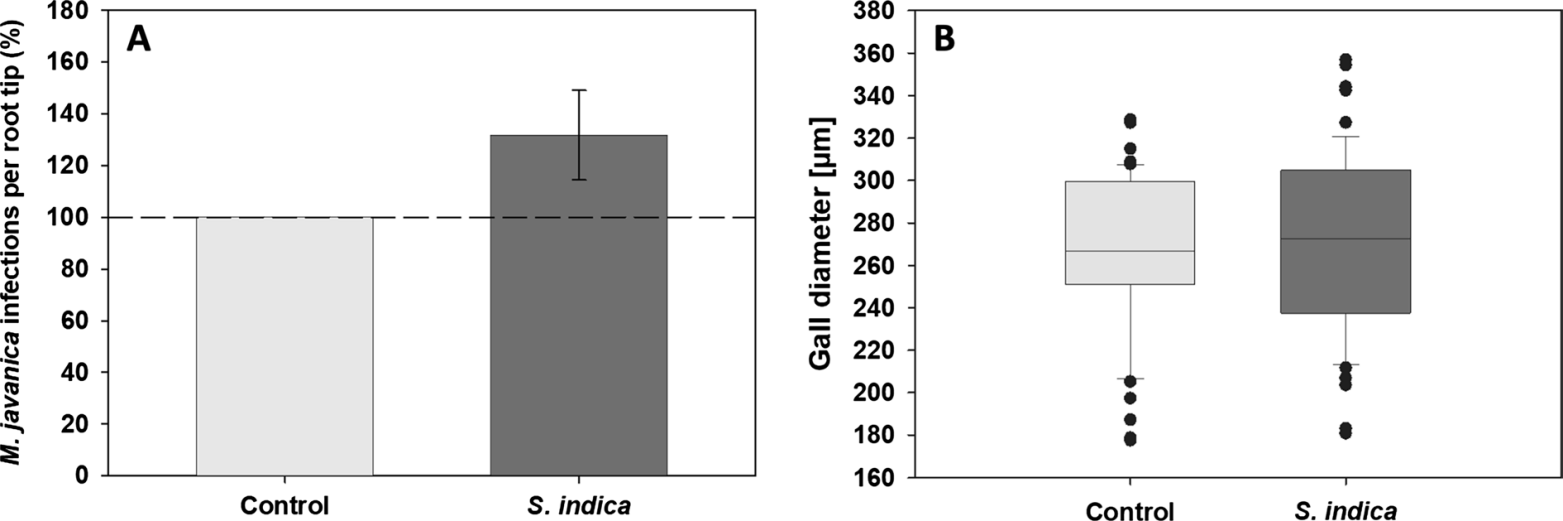
The indirect impact of *S. indica* colonization in split-root system on the development of root-knot nematode *M. javanica* (RKN). **A** The number of RKN galls in systemic roots of non-colonized plants and *S. indica-*colonized *A. thaliana* roots at 10 dai (RKN) (*n* = 89). Values indicate means ± SE of four independent repetitions. **B** Size of RKN galls in systemic roots of non-colonized plants and systemic roots (*n* = 110) of *S. indica-*colonized *A. thaliana* roots at 10 dai (RKN). Differences were analyzed using Student’s t test (*P* < 0.05) and are statistically non-significant

In RKN experiments, higher number of galls was observed in systemic roots of *S. indica*-colonized plants in comparison to systemic non-colonized roots at 10 dai (RKN) (Fig. 6A, + 31.8%), but the gall diameter was not affected by the fungal colonization (Fig. 6B).

### *S. indica* colonization changes systemic expression of defense-related genes

**Fig. 7:**
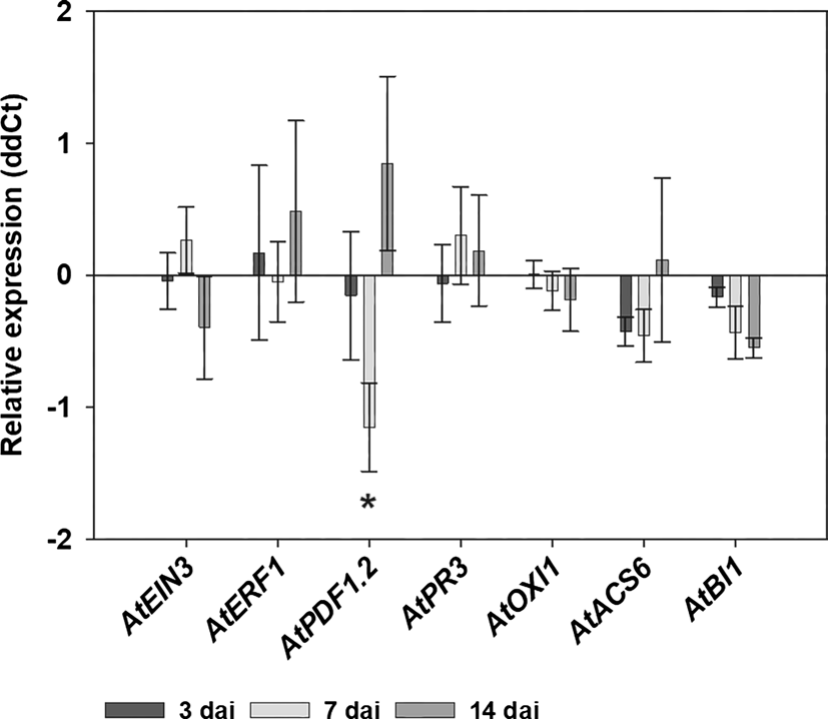
qPCR results showing the relative expression of genes involved in plant defense in systemic roots of *S. indica*-colonized plants in comparison to systemic roots of non-colonized plants 3, 7 and 14 dai with *S. indica*. Shown are means ± SE of three independent biological repetitions. Endogenous control: *AtUBP22*. Asterisk indicates significant difference (Student’s t test; *, *P* < 0.05)

The relative expression of plant defense-related genes *EIN3*, *ERF1*, *PDF1.2*, *OXI1*, *PR3*, *ACS6* and *BI1* was analyzed in systemic roots of *S. indica*-colonized plants in comparison to systemic roots of non-colonized plants at 3, 7 and 14 dai (*S. indica*). Only *PDF1.2* showed significant downregulation at 7 dai (*S. indica*) in systemic non-colonized half of the root with the second half colonized by *S. indica* (Fig. 7).

## Discussion

This work demonstrates that in systemic non-colonized part of the root, the levels of all measured sugars were less pronounced than in directly colonized roots as previously published (Opitz et al. 2021). Although they show a slight increment with respect to the non-colonized roots, the differences were not significant. This might be due to the different experimental set up as in three-chamber dishes the roots grew exclusively on the medium without sugar, whereas in dishes where the direct effects were analyzed, sugar might slightly diffuse from the upper shoot area containing sucrose, to the lower sucrose-free part. Furthermore, the amount of sucrose in directly colonized roots decreased significantly over time, from 3 to 14 dai, suggesting constant and increasing sucrose uptake by the growing endophyte. In contrast, in systemic non-colonized root part, we observed the opposite situation, the level of sucrose slightly increased from 3 to 14 dai. Moreover, these levels were generally slightly higher than those in the systemic roots of control non-colonized plants. This indicates that the fungal colonization triggers the accumulation of sucrose in the non-colonized, but also in the colonized part of the root. The measured concentrations indicate that sucrose gets transported in both parts of the root system however in unequal quantities. Our results, however, demonstrate that less sucrose was transported from the leaves to the roots without endophyte compared to the roots with *S. indica*. This suggests that the systemic endophyte-free half of the root in three-chamber dishes exhibited enhanced sink properties. These rather moderate changes in sugar pools correspond to insignificant alterations in the expression of sucrose synthases and invertases in systemic roots of *S. indica*-colonized plants. In contrast, directly colonized *A. thaliana* roots by *S. indica* (Opitz et al. 2021) and recently published data by de Rocchis et al. (2022a) on tomato roots directly colonized by *S. herbamans,* showed opposite patterns*.* The later report showed enhanced sugar levels as well as significant changes in expression of genes encoding enzymes involved in the metabolism of carbohydrate and sugar transport in the colonized roots. The differences between the local and systemic effects might be due, both to the lack of endophyte colonization in this part of the root, as well as to the lack of sucrose in the plant growing medium (Angeles-Nunez and Tiessen 2012; Wang and Ruan 2013).

Daneshkhah et al. (2013) showed the negative impact of *S. indica* on the development of the BCN *H. schachtii* in roots of *A. thaliana* that were colonized by the endophyte 3 days prior nematode inoculation (-3 dai). Encouraged by the CN data and the lack of information on RKN, we were interested to investigate the potential effect of *S. indica* colonization on the development of the RKN *M. javanica*. Therefore, we performed infection assays on endophyte-colonized *A. thaliana* plants that showed a significant reduction in the number of *M. javanica* galls at 10 dai. Interestingly, opposite to syncytia, the size of the galls between the colonized and non-colonized roots did not differ significantly.

**Fig. 8:**
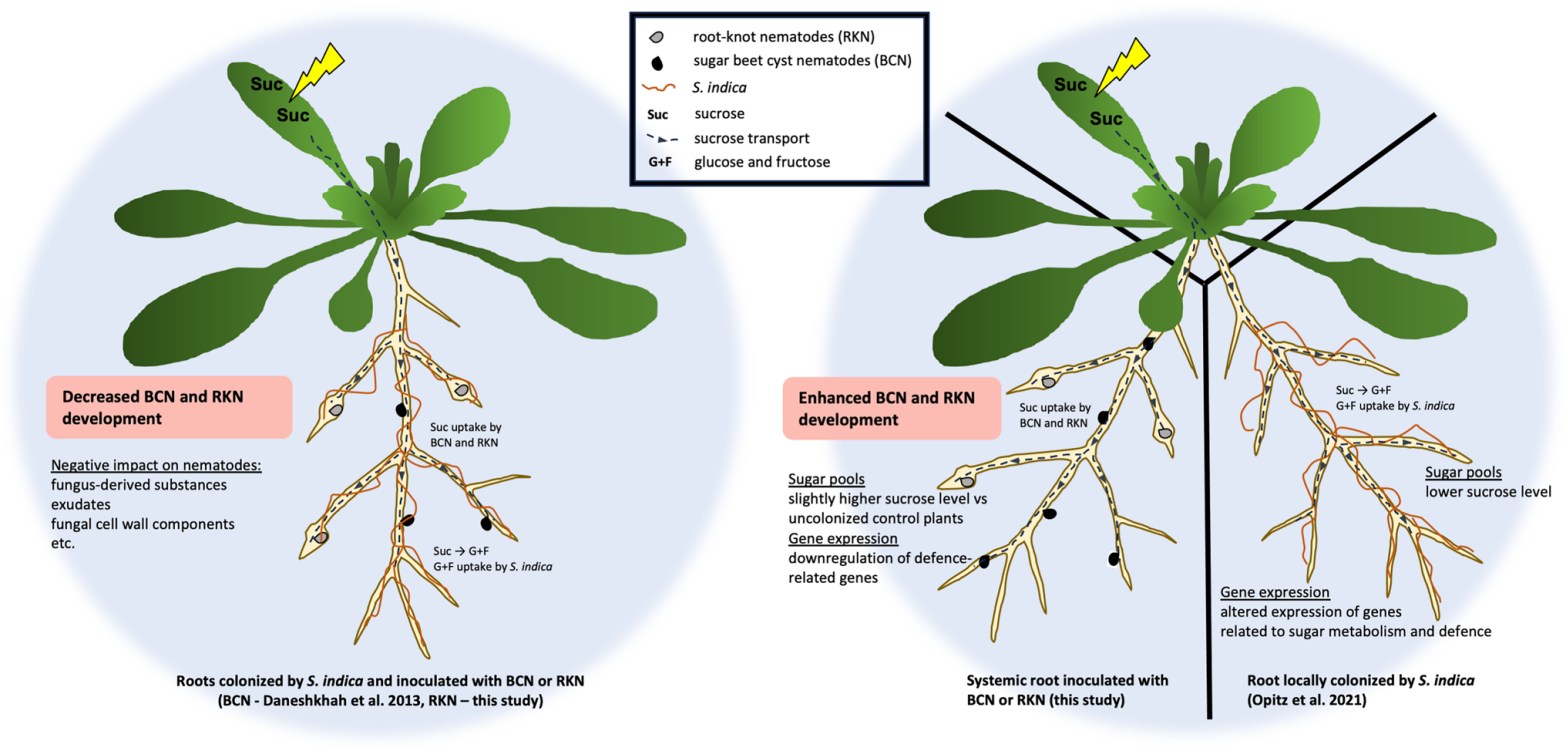
Schematic overview of interactions and cellular events during the tripartite association between host plant *A. thaliana*, fungal endophyte *S. indica* and the endoparasitic sugar beet cyst nematode *H. schachtii* (BCN) and root-knot nematode *M. javanica* (RKN). The events are depicted in roots concurrently colonized by the fungus and inoculated with nematodes (left panel; RKN in this study and BCN in Daneshkhah et al. 2013) as well as in in split-root system with one half of the root colonized by *S. indica* and the other half infected either with BCN or RKN (right panel; this study) and in root colonized by *S. indica* (right panel; Opitz et al. 2021), respectively

Although, in contrast to the directly colonized roots, the systemic alterations in sugar pools and gene expression were rather moderate, in the next step we used split-root system where one half of the root in one compartment of the three-chamber Petri dish was inoculated with *S. indica* and the other half with nematodes, *H. schachtii* or *M. javanica*, respectively. This set-up simulated the natural situation in soil where the plant root system is not evenly colonized by the endophyte. As controls we used plants half-inoculated with nematodes with the second half of the root mock-inoculated with the empty Käfer’s medium plug. This is the first report on split-root experiments involving *Serendipita* spp. and PPNs combinations. There are, however, few studies describing the impact of AMF *G. intraradices* on different nematode species. For instance, Elsen et al. (2008) using split-root system showed the AMF-induced biocontrol effects in banana plants against two species of migratory nematodes, *Pratylenchus coffeae* and *Radopholus similis*. Further, Vos et al. (2012a) using split-root system analyzed the effects on the migratory nematode *Pratylenchus penetrans* and the endoparasitic nematode *M. incognita* in tomato systemically inoculated with another AM fungi G. *mosseae.* Similar to experiments in banana, the authors reported the negative impact of AMF colonization on nematode development. The local AMF as well as systemic AMF triggered significant reduction in the number of nematode females, number of eggs as well as the gall index. All of these studies using the split-root system show the negative impact of the systemic colonization of fungal endophytes on the nematode development. In contrast to these reports, our results demonstrate significantly more females of *H. schachtii* in endophyte-free root part in three-chamber dishes in comparison to control plates. In addition, the length of syncytia in this part of the root was significantly smaller. This might suggest that these females are better supplied with sugars and they do not need to induce big feeding sites to get enough nutrients. Hence, there is a positive impact of systemic endophyte colonization on the general development of CN. In the case of RKNs, with *M. javanica*, we observed similar increase in the number of galls in comparison to the control plants. In addition, we did not see any differences in the gall size. This positive effect seems contradictory to many reports, as mentioned above. However, in some cases, the plants colonized by AMF can also support higher densities of nematodes due to their better nutrient status (Pettigrew et al. 2005; Bell et al. 2022) or/and suppressed root defense responses associated with resistance to invertebrate pests (Frew et al. 2018). There are only few reports demonstrating this phenomenon. For instance, Frew et al. (2018) showed that AMF colonization can reduce wheat biomass, while still having a positive nutritional impact on the host, and reduce the root defense compounds what results in promotion of population of migratory nematode *Pratylenchus neglectus*. Similar, the density of another Pratylenchid nematode, *P. thornei*, increased in mung bean plants inoculated with AMF (Gough et al. 2021). This increase was positively correlated with the increased amounts of phosphorus, zinc and copper in the shoots. Similarly, two recent studies report on positive effects on PPNs in potato plants colonized by AMF and infected by potato cyst nematode *Globodera pallida* in the glasshouse and under field conditions (Bell et al. 2022, 2023). In the infected plants, the majority of plant carbon was obtained by the nematodes while AMF maintained the transfer of nutrients. The authors conclude that in this situation, the resources acquired by AMF might not solely benefit the plant but also increase the host tolerance against the CNs that results in the host plant’s enhanced fitness. Hence, it is suggested that next to the frequent mycorrhizal-induced resistance (MIR; Cameron et al. 2013; Schouteden et al. 2015), there is also a mycorrhizal-induced tolerance (MIT) enabling the production of high biomass irrespective of the presence and success of nematodes. We observed a similar type of increased tolerance to nematodes in our work. In line with other reports, we demonstrate that the systemic non-colonized roots show slightly better nutritional status as well as suppressed defense responses, which might facilitate the development and parasitism of nematodes. Recently, we analyzed the expression of several genes involved in general defense responses in *A. thaliana* roots colonized directly by *S. indica* and concluded that the successful root colonization by *S. indica* depends on efficient suppression of plant immune responses (Daneshkhah et al. 2018). As shown here, in the non-colonized systemic roots in the split-root system, the majority of tested genes were not de-regulated as compared to the control plants. Only JA-responsive *PDF1.2* was significantly downregulated by the systemic *S. indica* colonization at the time around the nematode inoculation. Since PPNs actively downregulate its expression during infection as it was shown for the BCN (Siddique et al. 2011; Bennett et al. 2023) and RKNs (Bennett et al. 2023), this endophyte-triggered *PDF1.2* downregulation might facilitate better nematode development. The model demonstrating the interactions and cellular events that might be taking place during this tripartite association in directly colonized roots (Daneshkhah et al. 2013; Opitz et al. 2021) and nematode-infected systemic roots in three-chamber dishes (this study) is presented in Fig. 8.

The majority of reports highlight the positive biocontrol effects of fungal endophytes that additionally come along with enhancement of plant growth and yield. Recently, however, increasing number of studies show adverse impact of some root endophytes on plant growth as well as on pathogen control. Similarly, we demonstrate here that systemic root colonization of *S. indica* mimicking the natural situation in soil facilitates the development of CN and RKN in the non-colonized half of the root. This is most probably due to a slightly increased nutritional status and decreased plant defenses, which shows opposite behavior to that of the direct colonization of the fungus and nematodes within the same root system. Additional aspects such as altered phytohormone levels, changes in expression of many other host genes related to defense, sugar transport, etc. might play a role in this interaction and should be further investigated in the future. Nevertheless, our results shed first light on the other side of the coin, and rather unexplored aspect of the plant–endophyte relationship making clear that specific case-by-case studies covering different endophyte plant–pathogen combinations are necessary to evaluate the possible field application of different endophytes.

### Supplementary Information

Below is the link to the electronic supplementary material.

Supplementary file1 (PDF 108 KB)

## Data Availability

The datasets generated during the current study are available from the corresponding author on reasonable request.

## Abbreviations

AMF: Arbuscular mycorrhizal fungi
BCN: Sugar beet cyst nematode
CN: Cyst nematodes
dai: Days after inoculation
INV: Invertase
PPNs: Plant–parasitic nematodes
RKN: Root-knot nematodes
SUS: Sucrose synthase
