# Supplementary material for: The other side of the coin: systemic effects of Serendipita indica root colonization on development of sedentary plant–parasitic nematodes in Arabidopsis thaliana
Source: Planta. 2024 Apr 14;259(5):121. doi: 10.1007/s00425-024-04402-5 (PMC11016515; doi:10.1007/s00425-024-04402-5)
Supplement: Supplementary file 1 — Supplementary file1 (PDF 108 KB) [file 425_2024_4402_MOESM1_ESM.pdf]

Supplementary Table S1 Primer sequences used in this work

No.	Primer name	Sequence 5'—3'	Function
1	*AtUBP22 F*	ACAACATATGACCCGTTTATCGA	Endogenous control
2	*AtUBP22 R*	TGTTTAGGCGGAACGGATACT	
3	*AtSUS1 F*	GCGCGTCCACAGCCAACGTG	Sucrose synthase
4	*AtSUS1 R*	ACCAGGCCTTGGCCTCACAGC	
5	*AtSUS2 F*	AGGGTGTACCAAATCTCAT	Sucrose synthase
6	*AtSUS2 R*	CATAGTGAAAGCTGTGTGG	
7	*AtSUS3 F*	GAGCACGGGCTCTCGGGTTT	Sucrose synthase
8	*AtSUS3 R*	GCCGAGTCTCACGACGCTCC	
9	*AtSUS4 F*	CACACTTCCCGGGTTGTACCGT	Sucrose synthase
10	*AtSUS4 R*	GCGCAAGCGAGTGTTCTTACCG	
11	*AtSUS5 F*	GTCCTCGAAGCTCGGAGGGC	Sucrose synthase
12	*AtSUS5 R*	CTCCCTGCGCTTTCTCCCCA	
13	*AtSUS6 F*	CGCCTTGATTGCAAGCCAGACC	Sucrose synthase
14	*AtSUS6 R*	TGGCCTGTCCTTGCTTCCTGC	
15	*AtCINV1 F*	TCGAGGGCCATGAGTGGCGC	Cytosolic invertase
16	*AtCINV1 R*	CGCGCGTCTTGCGATCTGCG	
17	*AtCINV2 F*	CGGTGGATCTTGGCCAGTATTGC	Cytosolic invertase
18	*AtCINV2 R*	CCAGCAATCTCGGTGTAGCCGT	
19	*AtEIN3 F*	CATTTCTCCAGGTTACAATGAT	Transcription factor of AtERF1
20	*AtEIN3 R*	AGCTTGTGGAACAGGAC	
21	*AtERF1 F*	CGGCGGAGAGAGTTCAAGAGTC	Activator of AtPDF1.2
22	*AtERF1 R*	TCCCACTATTTTCAGAAGACCCC	
23	*AtPDF1.2 F*	CTGCTTTCGACGCACCGGCAA	Plant defense marker gene for JA/ET pathway
24	*AtPDF1.2 R*	ACCCCTGACCATGTCCCACTTGG	
25	*AtOXI1 F*	TCATCTACATTGGCCGTGTC	Protein kinase required for activation of AtMPK6
26	*AtOXI1 R*	CGTCGCTCCATACAACATCT	
27	*AtACS6 F*	CCGGGAATGTTTGAAGTCTCTTG	Substrate for AtMPK6
28	*AtACS6 R*	CGGTCTTAAGTCTGTGCACGG	
29	*AtPR3 F*	ATCACCGCTGCAAAGTCCTTC	Pathogenesis related gene (chitinase)
30	*AtPR3 R*	TGCTGTAGCCCATCCACCTG	
31	*AtBI1 F*	GCAGCAGCAATGTTAGCAAG	Attenuator of cell death
32	*AtBI1 R*	CACCACCATGTATCCCACAA	
33	*SiTEF F*	ATCGTCGCTGTCAACAAGAT	Elongation factor 1 alpha
34	*SiTEF R*	ACCGTCTTGGGGTTGTATCC	

Supplementary Table S2 Relative gene expression (ddCt) of *AtSUS* and *AtCINV* genes in systemic roots of *S. indica*-colonized *A. thaliana* plants in comparison to non-colonized controls (*n*=3; ± SE)

genes	Systemic roots of *S. indica*-colonized plants		
	**3 dai**	**7 dai**	**14 dai**
AtSUS1	-0.12 (±0.13)	0.80 (±0.31)	-0.21 (±0.36)
AtSUS2	-0.09 (±0.33)	-0.51 (±0.31)	-0.26 (±0.24)
AtSUS3	0.16 (±0.55)	-0.19 (±0.09)	-0.31 (±0.33)
AtSUS4	-0.11 (±0.41)	0.49 (±0.25)	1.19 (±0.43)
AtSUS5	0.38 (±0.54)	-0.08 (±0.26)	-0.53 (±0.13)
AtSUS6	-0.14 (±0.39)	0.00 (±0.17)	-0.62 (±0.33)
AtCINV1	0.00 (±0.34)	0.27 (±0.21)	-0.33 (±0.30)
AtCINV2	0.10 (±0.37)	0.59 (±0.35)	-0.60 (±0.24)
